# Impact of cigarette toxicants on sarcopenic obesity: An in silico network toxicology and molecular dynamics study

**DOI:** 10.18332/tid/226887

**Published:** 2026-09-05

**Authors:** Zijing Li, Daoyuan Li, Yuli Huang, Yushang Liu, Xinye Ouyang, Yufei Xu, Wenjuan Wu, Yanbiao Zhong, Maoyuan Wang

**Affiliations:** 1 Department of Rehabilitation Medicine, First Affiliated Hospital of Gannan Medical University, Ganzhou City, China; 2 School of Basic Medical Sciences, Gannan Medical University, Ganzhou City, China; 3 School of Rehabilitation and Sports Health, Gannan Medical University, Ganzhou City, China; 4 Department of Rehabilitation Medicine, The Third Affiliated Hospital, Sun Yat-sen University, Guangzhou City, China; 5 The First Clinical Medical College, Gannan Medical University, Ganzhou City, China; 6 Ganzhou Key Laboratory of Rehabilitation Medicine, First Affiliated Hospital of Gannan Medical University, Ganzhou City, China

**Keywords:** molecular dynamics simulation, molecular docking, nicotine, sarcopenia obesity, coal tar

## Abstract

**INTRODUCTION:**

Sarcopenic obesity (SO) is a geriatric metabolic syndrome characterized by muscle mass loss, muscle strength decline, and excessive fat accumulation. Nicotine and coal tar disrupt skeletal muscle homeostasis and lipid metabolism. This study aims to elucidate the relevant molecular mechanisms by which nicotine and coal tar trigger sarcopenic obesity using an integrated in silico approach.

**METHODS:**

This in silico study was conducted in May 2026 using publicly available databases and computational platforms. Targets for nicotine, coal tar, and SO were retrieved from GeneCards and OMIM, and the intersection of targets was identified using Venny. KEGG enrichment was performed to detect key pathways. A protein-protein interaction (PPI) network was constructed via STRING, and the top 10 core genes were ranked by the Maximal Clique Centrality (MCC) algorithm in Cytoscape. Three GEO transcriptomic datasets (GSE290570, GSE262419, GSE226045) were used to cross-validate differentially expressed genes related to nicotine, coal tar, and SO. Molecular docking between nicotine and five core proteins was performed using CB-DOCK2, and 50 ns molecular dynamics simulations with GROMACS were used to assess complex stability via root-mean-square deviation (RMSD), root-mean-square fluctuation (RMSF), radius of gyration (Rg), solvent-accessible surface area (SASA), and hydrogen-bond analysis.

**RESULTS:**

Twenty intersection targets were identified. Enriched pathways included MAPK, PI3K/Akt, p53, cholesterol metabolism, and ferroptosis. Five core genes (IL1β, IGF1, FGF2, CTNNB1, and CXCL12) were cross-validated by GEO transcriptomic data. Nicotine bound stably to CTNNB1, CXCL12, IGF1, and IL1β with binding energies ranging from -3.3 to -4.2 kcal/mol, whereas FGF2 showed a positive value (0.7). MD simulations confirmed that all complexes maintained structural stability throughout the 50 ns trajectory.

**CONCLUSIONS:**

Nicotine and coal tar may regulate core genes and inflammation- and metabolism-related pathways to promote SO progression. This study provides theoretical references for preventing tobacco exposure-induced SO.

## INTRODUCTION

Sarcopenic obesity (SO) is a high-risk geriatric metabolic syndrome characterized by skeletal muscle mass loss, muscle strength decline, and excessive fat accumulation^1^. In 2022, the European Society for Clinical Nutrition and Metabolism (ESPEN) and the European Association for the Study of Obesity (EASO) released an authoritative expert consensus that explicitly defines SO as an obese state accompanied by reduced skeletal muscle mass, muscle strength, or physical function^2^. Against the backdrop of global population aging and a continuous rise in obesity prevalence, the overall prevalence of SO in the elderly has reached 11%, fluctuating from 2.75% to 20% due to discrepancies in diagnostic criteria. The incidence of SO is nearly twice that of simple obesity, rendering it a critical public health threat to the health of older adults^3,4^. Compared with isolated sarcopenia or simple obesity, SO exerts a significant synergistic pathological damaging effect, markedly elevating the risks of metabolic disorders, motor function deterioration, falls, disability, and all-cause mortality. Epidemiological data indicate that SO increases the mortality risk by 24% and imposes a heavy socioeconomic burden on the global healthcare system^5,6^. Pathogenically, SO is not a simple combination of sarcopenia and obesity; instead, it represents a complex metabolic disorder driven by a mutually promotional and vicious cycle between the two conditions. Obesity triggers chronic low-grade inflammation, oxidative stress, and insulin resistance, which inhibit muscle protein synthesis and enhance protein catabolism, thereby directly inducing myocyte apoptosis and muscular atrophy. Conversely, the loss of muscle mass and strength further reduces energy consumption and exacerbates fat accumulation, forming a vicious loop of fat accumulation-inflammation activation-muscle loss-progressive fat gain^7^. Additionally, mitochondrial dysfunction, energy metabolism imbalance, nutritional imbalance, and endocrine disorders collectively participate in the onset and progression of SO, ultimately leading to muscular functional decline and the collapse of systemic metabolic homeostasis. Given the lack of unified diagnostic criteria and specific intervention strategies for SO currently, exploring its risk factors and molecular regulatory mechanisms is of important clinical significance^8^.

Smoking is a well-recognized global public health hazard. Numerous toxic and harmful substances released by tobacco combustion invade multiple human systems and organs, inducing a spectrum of severe health damages including tumors, cardiovascular and cerebrovascular diseases, respiratory diseases, and metabolic disorders^9^. Epidemiological and basic studies have suggested that smoking serves not only as a core risk factor for chronic non-communicable diseases but also profoundly disturbs the homeostasis of skeletal muscle mass, lipid metabolism, and bone metabolism, closely associated with muscle loss and abnormal fat deposition^10,11^. Tobacco smoke contains thousands of chemical components, among which nicotine and coal tar are the predominant toxic and addictive constituents. These substances circulate throughout the body via the blood and target multiple organs including skeletal muscle, adipose tissue, liver, and neuroendocrine system, regulating cell proliferation, apoptosis, inflammatory response, and energy metabolism^12^. Accumulating evidence demonstrates that nicotine modulates the balance of protein synthesis and degradation by activating nicotinic acetylcholine receptors, while polycyclic aromatic hydrocarbons and other components in coal tar induce oxidative stress and chronic low-grade inflammation. Notably, chronic inflammation, mitochondrial dysfunction, imbalanced protein metabolism, and aberrant adipocyte differentiation constitute the core pathological links of SO progression^13^. Relevant toxicological studies verified that tobacco toxic components such as nicotine disrupt the metabolic homeostasis of the musculoskeletal system via specific targets and signaling pathways, triggering abnormal bone metabolism and skeletal muscle injury. These findings provide a theoretical basis for the pathogenic role of tobacco exposure in musculoskeletal metabolic diseases^14^. Nevertheless, it remains unclear whether nicotine and coal tar synergistically regulate muscle loss and fat accumulation through multi-target and multi-pathway networks, and the molecular regulatory landscape underlying SO induced by their combined exposure is yet to be elucidated.

Accordingly, this study adopted an integrated analytical strategy combining network toxicology, molecular docking, and molecular dynamics simulation. We screened the active targets of nicotine and coal tar as well as disease targets of SO, constructed a toxicant-target-disease interaction network, and identified core regulatory genes and key signaling pathways. Meanwhile, functional enrichment analysis was performed to clarify the biological processes and molecular mechanisms by which exposure to nicotine and coal tar induces SO. This study aims to reveal the toxic mechanisms of nicotine and coal tar in triggering SO, and provide a theoretical basis for the prevention and control of tobacco exposure-related diseases.

## METHODS

Supplementary file Figure S1 illustrates the workflow for investigating nicotine- and coal tar-induced sarcopenic obesity via network toxicology, molecular docking, and molecular dynamics simulation

### Screening of intersection targets of nicotine, coal tar and SO and KEGG pathway enrichment analysis

Based on a comprehensive literature review of cigarette smoke composition, we identified nicotine and tobacco tar as the two core toxicants for investigation. Tobacco tar, also referred to as cigarette smoke condensate, is a complex mixture of thousands of compounds generated during tobacco combustion and is recognized as one of the most hazardous fractions of cigarette smoke. Consistent with previous network toxicology studies on cigarette-related diseases that collectively refer to tobacco tar as ‘coal tar’, we adopted this terminology. Toxicological data and chemical structures for nicotine and tobacco tar were retrieved from the PubChem database^15^. To identify potential targets of these toxicants in SO, genes associated with nicotine, tobacco tar, and SO were retrieved from the GeneCards and OMIM databases using a correlation score >1 as the screening threshold. After removing duplicates from the three gene sets, the overlapping targets among nicotine, tobacco tar, and SO were identified using the Venny 2.1 online tool. For functional annotation of the intersecting targets, KEGG pathway enrichment analysis was performed via the DAVID database with ‘Homo sapiens’ as the species setting and p<0.05 as the significance threshold. The enrichment ratio was used to rank the pathways, and the results were visualized as bar charts^16^.

### Construction of PPI network and screening of core hub genes

The obtained intersection targets were uploaded to the STRING database with ‘Homo sapiens’ restricted and a minimum interaction score ≥0.4 to construct the PPI network. Isolated nodes were removed to ensure network reliability. The network files exported from STRING were imported into Cytoscape software for visualization and topological analysis. The MCC algorithm in the CytoHubba plugin was applied to rank network nodes, and the top 10 ranked genes were selected as core targets for subsequent molecular docking and molecular dynamics simulation^17^.

### Acquisition of transcriptomic data and verification of differentially expressed genes

Three sets of public transcriptomic datasets were downloaded from the NCBI GEO database for cross-validation: GSE290570 contained transcriptomic data of aged mice with SO induced by a 60% kcal high-calorie diet and a normal diet control (12% kcal); GSE262419 comprised TempOSeq data of human iPSC-differentiated cardiomyocytes treated with nicotine; GSE226045 included transcriptomic data of human skin/epithelial tissues exposed to coal tar. The gProfiler platform was used for homologous conversion between mouse and human genes to unify the gene annotation system. DESeq2 was employed for differential expression analysis between the nicotine-treated group and vehicle (VEH) control group in GSE262419. Genes with |log2FC| >0.5 and adjusted p<0.05 were defined as significantly differentially expressed genes (DEGs). The expression matrix was subjected to CPM normalization, log2(CPM+1) transformation, and row-wise Z-score standardization. The SO-, nicotine-, and coal tar-related target genes were intersected with DEGs from corresponding datasets to obtain cross-disease-toxicant targets, based on which a regulatory network of cigarette toxicants in SO was established. Heatmaps of all DEGs, top 50 hub genes, and box plots of key genes were plotted based on the standardized expression matrix to verify the expression trends of hub genes under nicotine stimulation^15^.

### Molecular docking for binding affinity verification between core targets and nicotine

Considering that coal tar is a multicomponent mixture, nicotine was chosen as a pure monomer for the subsequent molecular docking and dynamics simulation. Five core proteins (CTNNB1, CXCL12, FGF2, IGF1, IL1β) selected from the top 10 hub genes were subjected to molecular docking with nicotine; the top five targets were prioritized due to their high structural resolution. The 2D structure of nicotine was retrieved from the PubChem database; the crystal structures of the five target proteins were downloaded from the RCSB PDB database. The CB-DOCK2 online blind docking platform automatically completed receptor hydrogenation, missing atom repair, charge calculation, and active pocket identification without manual parameter setting. Based on the built-in AutoDock Vina algorithm, the optimal binding conformation was screened, and the binding free energy was calculated to evaluate the binding affinity and structural stability between molecules. Only binding energy was used to assess the binding capacity in this study, without 3D interaction mapping^18^.

### Molecular dynamics simulation

To further verify the conformational stability of nicotine-target protein complexes obtained from molecular docking under physiological conditions, 50 ns all-atom molecular dynamics simulations were performed on five complexes using GROMACS 2022 software. The AMBER99SB-ILD force field was adopted for protein receptors, the GAFF force field was matched for nicotine ligands via Antechamber, and the TIP3P water model was applied for the aqueous environment. The complexes were placed in a cubic periodic water box with a 1 nm buffer layer, and counterions were added to neutralize the system charge. Simulations were conducted under the NPT ensemble at 300 K and 1 bar with an integration time step of 2 fs. The PME algorithm was used for long-range electrostatic interactions, and chemical bonds were constrained by the SHAKE algorithm. Simulation trajectories were analyzed to calculate RMSD, RMSF, Rg, SASA, and intramolecular hydrogen bond numbers. The dynamic structure and binding stability of each complex were comprehensively evaluated from the perspectives of conformational stability, residue flexibility, protein compactness, solvent exposure characteristics, and intramolecular interactions^19^.

## RESULTS

### Screening of intersection targets and KEGG pathway enrichment analysis

Venn diagram analysis identified 3712 nicotine-related targets, 90 coal tar-related targets, and 146 SO-related targets. Among them, 121 common targets overlapped between nicotine and coal tar, 50 between nicotine and SO, 5 between coal tar and SO, and a total of 20 intersection targets were shared by all three (Supplementary file Figure S2A).

KEGG pathway enrichment analysis yielded multiple significantly enriched pathways. Highly enriched pathways included core signaling pathways governing muscle metabolism and cellular homeostasis, such as the MAPK, PI3K/Akt, and p53 signaling pathways. Pathways closely associated with lipid metabolism and systemic energy disorders, including cholesterol metabolism and fat digestion and absorption, were also enriched. In addition, pathways related to chronic inflammation, such as inflammatory bowel disease and ferroptosis, and muscular atrophy were enriched. These pathways collaboratively regulate muscle mass maintenance, lipid metabolic balance, and inflammatory response, playing roles in the pathogenesis and progression of SO induced by nicotine and coal tar (Supplementary file Figure S2B).

### Construction of PPI network and screening of core hub genes

The 20 intersection targets were used to construct the PPI network (Supplementary file Figure S3A). After Cytoscape visualization and node ranking via the MCC algorithm in CytoHubba, the top 10 core genes were IL1β, IGF1, FGF2, CTNNB1, CXCL12, CCL2, PTGS2, ESR1, MMP3, and MMP1 (Supplementary file Figure S3B). These genes exhibited the highest connectivity and betweenness centrality in the network, suggesting that nicotine and coal tar may disrupt muscle-adipose metabolic balance by regulating these core genes, ultimately inducing SO.

### Verification of differentially expressed genes based on GEO transcriptomic datasets

Differential expression analysis of GSE262419 between the nicotine-treated group and VEH control group identified 1686 significant DEGs under the criteria of |log2FC| >0.5 and adjusted p<0.05. The global DEG heatmap showed that gene expression profiles were clearly clustered between the two groups, with high intra-group consistency and distinct inter-group differentiation. The hub gene heatmap revealed significant expression differences of the top 50 hub genes between the two groups (Supplementary file Figures S4 and S5).

Among the top 10 core targets, CCL2, CXCL12, PTGS2, MMP3, IGF1, and ESR1 were upregulated upon nicotine intervention, while IL1β, FGF2, CTNNB1, and MMP1 were downregulated. Box plots further intuitively presented the expression distribution differences of core target genes under nicotine stimulation, validating their expression alteration patterns. Cross-analysis of three transcriptomic datasets showed significant overlap of DEGs related to nicotine, coal tar, and SO, indicating potential molecular regulatory correlations among the three.

### Molecular docking

Molecular docking results showed that the optimal binding free energies of nicotine to CTNNB1, CXCL12, IGF1, and IL1β were -4.2, -4.1, -3.9, and -3.3 kcal/ mol, respectively, indicating that nicotine could form stable binding conformations with the four target proteins (Supplementary file Figure S6). In contrast, the binding energy of nicotine to FGF2 was positive at 0.7 kcal/mol. Structural and physicochemical analyses revealed the underlying mechanism: the surface of FGF2 is rich in positively charged lysine and arginine residues, which preferentially bind to natural negatively charged ligands under physiological conditions. Nicotine is easily protonated and positively charged at physiological pH, leading to electrostatic repulsion with the electropositive surface of FGF2. Moreover, the natural active pocket of FGF2 is structurally adapted to macromolecular proteins or glycans, with poor spatial matching to small-molecule nicotine, failing to form sufficient hydrophobic contacts and hydrogen bonds to offset the entropy loss during ligand binding. Furthermore, the default rigid receptor docking mode of CB-DOCK2 cannot simulate conformational rearrangement induced by side-chain fitting, further resulting in no energetically favorable binding site being screened out. The positive binding energy suggested that nicotine could not spontaneously form a thermodynamically stable complex with FGF2.

### Molecular dynamics simulation

Molecular dynamics results demonstrated that the backbones of all five nicotine-target protein complexes gradually converged and maintained structural integrity during the 50 ns simulation. Key kinetic parameters such as RMSD and RMSF fluctuated within normal physiological ranges, indicating favorable overall conformational stability. The FGF2-nicotine complex presented the lowest backbone RMSD of 0.095 nm, with minimal skeletal fluctuation and the most compact spatial conformation. CTNNB1, with a longer sequence and larger molecular weight, showed relatively higher RMSD and radius of gyration, maintaining its spatial structure mainly via intramolecular hydrogen bonds. The kinetic curves of CXCL12, IGF1, and IL1β complexes changed gently with residue flexibility within physiological ranges, reflecting stable system conformation.

Combined with molecular docking results, nicotine lacks thermodynamic advantages for spontaneous binding to FGF2 under rigid docking conditions and cannot form stable initial complexes via free diffusion. However, molecular dynamics simulations reflected protein flexibility; conformational rearrangement via side-chain-induced fitting, solvation effects, and weak non-covalent interactions compensated for energy defects, enabling the pre-assembled FGF2-nicotine complex to maintain conformational homeostasis in physiological aqueous environments (Supplementary file Figure S7). Collectively, nicotine can spontaneously form stable binding systems with CTNNB1, CXCL12, IGF1, and IL1β. It cannot autonomously target and bind FGF2 but maintains conformational stability mediated by the *in vivo* microenvironment and auxiliary factors, participating in the pathological regulation of SO indirectly via signaling pathway networks.

## DISCUSSION

This study integrated multiple bioinformatic approaches to explore the molecular mechanisms of SO induced by nicotine and coal tar exposure. As the principal toxic components of tobacco, nicotine and coal tar accumulate in skeletal muscle and adipose tissue, disrupting muscle remodeling and glycolipid metabolic homeostasis and severely endangering the health of the elderly. Existing studies have confirmed that exposure to the two substances induces chronic inflammation and oxidative stress, leading to muscle mass loss, muscle strength decline, and abnormal fat accumulation^20^. Nicotine and coal tar can stably bind to core targets including IL1β, IGF1, CXCL12, and CTNNB1, regulating key signaling pathways such as MAPK, PI3K/Akt, and p53 to form a multi-target and multi-pathway regulatory network, thereby promoting the occurrence and progression of SO. At present, the molecular mechanisms underlying the synergistic effect of nicotine and coal tar in mediating SO remain poorly understood. This study resolved the regulatory mechanisms via a multi-technique integrated strategy, aiming to provide a theoretical basis for screening therapeutic targets for tobacco exposure-induced SO. Based on the screened core targets, the roles and underlying molecular mechanisms of each key gene in sarcopenic obesity induced by nicotine and coal tar were further elaborated, respectively.

The Wnt/β-Catenin signaling pathway mediated by CTNNB1 (β-catenin) is inhibited by STAT3 in sarcopenia. Reduced activity of this pathway suppresses myoblast proliferation and differentiation and accelerates sarcopenia progression, while STAT3 blockade restores β-catenin signaling function and enhances skeletal muscle regeneration and muscle mass^21^. Meanwhile, β-catenin regulates obesity-related signaling networks, and its signaling disorder drives obesity and concomitant inflammatory responses. Nicotine can activate the Wnt/β-Catenin pathway, which also serves as a critical mediator of nicotine-regulated cellular biological behaviors under high-glucose conditions. Collectively, nicotine may intervene in myogenesis and lipid metabolism by activating the Wnt/β-Catenin pathway mediated by β-catenin, thereby participating in the pathological evolution of SO^22^.

CXCL12, also known as stromal cell-derived factor 1, is a key molecule regulating skeletal muscle development. It protects against muscle injury by modulating muscle satellite cell function and mediating muscle regeneration. Specifically, CXCL12 inhibits excessive STAT3 activation and upregulates Pax7 expression to maintain satellite cell homeostasis and guarantee the regenerative capacity of skeletal muscle stem cells. Imbalance of this signaling axis is also a major inducement of sarcopenia^23^. Research demonstrated that nicotine targets the α7 subunit of nicotinic acetylcholine receptors and downregulates CXCL12 expression. Inhibited CXCL12 expression disrupts the downstream STAT3/Pax7 signaling axis and normal satellite cell function, ultimately facilitating the development of sarcopenia and other muscle-related disorders^24^.

FGF2 is an essential regulator of skeletal development and muscle growth. Physiologically, it activates the proliferation of skeletal muscle satellite cells and promotes muscle tissue regeneration; nevertheless, aberrantly elevated FGF2 continuously depletes satellite cell reserves and eventually leads to muscle mass reduction. FGF2 is also involved in skeletal muscle repair and lipid metabolism regulation, and imbalanced expression and signaling of FGF2 simultaneously trigger muscle loss and abnormal fat accumulation, driving the progression of SO^23^. Relevant studies have verified that nicotine significantly upregulates FGF2 expression in wound tissues, suggesting that nicotine may disrupt the physiological function of FGF2 by elevating its expression and ultimately participate in the pathological process of SO^25^.

IGF1 is mainly secreted by the liver, closely correlated with human aging and physiological functional changes, and modulates systemic metabolic homeostasis. Its expression level is associated with muscle mass and strength, and skeletal muscle can reversibly regulate IGF1 secretion^26^. Abnormal IGF1 secretion disturbs lipid metabolism, causes excessive fat accumulation, and impairs skeletal muscle structure and function. Intestinal-derived interleukin-13 promotes hepatic IGF1 secretion, and imbalanced IGF1 expression disrupts the homeostasis of muscle proliferation and lipid metabolism, triggering muscle loss and excessive fat deposition, and facilitating the onset of sarcopenia and obesity^27^. These findings indicate that nicotine may participate in the pathological progression of SO by targeting IGF1.

Nicotine upregulates thioredoxin-interacting protein expression, activates inflammasomes, and promotes IL-1β release. Activated inflammasomes facilitate the maturation and secretion of IL-1β^28^. As a pivotal pro-inflammatory cytokine, IL-1β is overexpressed along with obesity-induced inflammation, triggering insulin resistance. It also interferes with myofiber differentiation and muscle protein metabolism, accelerating protein degradation and muscle mass attenuation. Muscle loss further exacerbates insulin resistance and promotes abnormal lipid accumulation, jointly mediating the onset and progression of SO^29^.

KEGG pathway enrichment analysis revealed that nicotine, coal tar, and SO were significantly enriched in the MAPK, PI3K/Akt, and p53 pathways, all of which are inflammation-related signaling pathways. These findings provide critical evidence for elucidating the inflammatory mechanisms of SO and related musculoskeletal diseases induced by major toxic tobacco components. Chronic systemic inflammation induced by obesity and metabolic syndrome activates the MAPK signaling pathway. Persistent abnormal activation of MAPK disrupts skeletal muscle structure and function, accelerates the progression of SO and musculoskeletal disorders, and is also implicated in age-related sarcopenia^30^. In sarcopenia models, abnormal MAPK activation combined with decreased PI3K pathway activity synergistically induces muscular atrophy. Lactoferrin combined with creatine can regulate the two pathways, inhibit aberrant signaling, restore pathway activity, and ameliorate muscle loss^31^.

Aberrant activity of the PI3K/Akt pathway is closely associated with sarcopenia, obesity, and SO, with differential regulatory effects. In SO, inflammatory factors and free fatty acids released by adipose tissue inhibit the PI3K/Akt pathway, triggering insulin resistance and anabolic resistance, thereby causing muscle loss and fat accumulation and aggravating disease severity^32^. Pathway inhibition also directly exacerbates skeletal muscle loss and promotes sarcopenia progression. In contrast, excessive activation of the PI3K/Akt pathway accelerates preadipocyte differentiation, enhances lipid synthesis and accumulation, induces adipose hyperplasia, disrupts glycolipid metabolism, and triggers insulin resistance, ultimately leading to obesity and related metabolic disorders. Exercise can reactivate the PI3K/ Akt pathway via crosstalk between myokines and adipokines, promote muscle protein synthesis, improve metabolic status, and alleviate SO. In addition, food-derived bioactive peptides can activate the PI3K/Akt pathway to exert anti-sarcopenia effects^33,34^.

Obesity induces cellular senescence and activates the p53 signaling pathway. Excessively activated p53 aggravates cellular senescence, promotes inflammatory factor secretion, disturbs systemic metabolism, and impairs cardiac function. Clearance of senescent cells reverses p53-mediated pathological alterations and ameliorates obesity-related metabolic and cardiac damage. Meanwhile, aging-related metabolic disorders and chronic inflammation also activate the p53 pathway^35^. Activated p53 upregulates senescence-related genes, induces myocyte cell cycle arrest, senescence, and apoptosis, disrupts muscle protein metabolic homeostasis, amplifies inflammatory responses, disturbs immune balance, inhibits muscle repair and regeneration, and causes progressive skeletal muscle damage, driving sarcopenia progression. Collectively, the p53 pathway is involved in the pathological processes of both obesity and sarcopenia, playing a crucial role in the development of SO^36^.

### Strengths and limitations

This study has three main strengths. First, an integrated multi-dimensional analytical system combining network toxicology, transcriptomic validation, molecular docking, and molecular dynamics simulation was established, breaking the limitations of single research methods and enabling systematic and comprehensive mechanistic interpretation from target screening and expression verification to molecular conformational stability analysis. Second, focusing on the real smoking exposure characteristics of the population, this study explored the synergistic toxic effects of nicotine and coal tar, which is more consistent with actual pathological exposure scenarios than single toxicant research, rendering the screened core targets and regulatory networks higher biological reference value. Third, cross-validation via GEO transcriptomic datasets combined with atomic-level verification of target binding capacity and complex stability using molecular docking and dynamics simulation achieved mutual confirmation from multiple dimensions, effectively improving the reliability of research conclusions.

Several limitations of the present study should be acknowledged. First, this study is primarily based on bioinformatics prediction and computational simulation, with a lack of *in vitro* and *in vivo* experimental validation. The mechanistic exploration was conducted through public database mining, GEO transcriptomic profiling, molecular docking and molecular dynamics simulation, while cellular and animal functional experiments were not performed. The identified hub genes, key signaling pathways, and toxin-target binding patterns are merely bioinformatic predictions. Without validations including gene intervention, protein quantification, pathological staining, and animal phenotypic assessment, it remains difficult to confirm the actual regulatory effects and causal relationships of nicotine and coal tar on sarcopenic obesity at the cellular and organismal levels. Second, gradient designs for toxicant exposure dose and intervention duration were absent, leading to insufficient analysis of dose-effect relationships and temporal regulatory characteristics. This study only explored the molecular associations of nicotine and coal tar with sarcopenic obesity at a holistic level. No exposure models with different concentrations and treatment durations were established, which fails to clarify the toxicological disparities among various doses and delineate the temporal patterns underlying the onset and progression of toxicant-induced sarcopenic obesity. Third, tissue-specific differences were not fully considered, and independent mechanistic interpretation across distinct tissues is lacking. Sarcopenic obesity is pathologically characterized by concomitant skeletal muscle atrophy and aberrant adipose accumulation. Nevertheless, this study only performed global gene network analysis, without comparing the differential gene expression and pathway activation signatures between skeletal muscle and adipose tissue. Accordingly, the tissue-specific regulatory mechanisms exerted by nicotine and coal tar cannot be well elucidated.

## CONCLUSIONS

Based on an integrated computational research system combining network toxicology, molecular docking, and molecular dynamics simulation, coupled with mining of public bioinformatics databases, this study preliminarily predicted and elucidated that nicotine and coal tar can synergistically regulate the pathological progression of sarcopenic obesity via a multi-target and multi-signaling pathway pattern. As a mechanistic exploration merely at the level of bioinformatics prediction and computational simulation, this study provides certain theoretical references for further research on the correlation between tobacco exposure and sarcopenic obesity.

## Supplementary Material



## Data Availability

All data used in this study were obtained from publicly available databases, including GeneCards (https://www.genecards.org/accessed on 12 May 2026), OMIM (https://www.omim.org/accessed on 12 2026), Venny (https://bioinfogp.cnb.csic.es/tools/venny/accessed on 12 May 2026), DAVID (https://davidbioinformatics.nih.gov/accessed on 13 May 2026), the microbiome visualization cloud platform (https://www.bioinformatics.com.cn/accessed on 13 May 2026) STRING (https://cn.string-db.org/accessed on 13 May 2026), RCSB PDB (https://www.rcsb.org/pages/about-us/index/accessed on 14 January 2026) CB Dock2 (https://cadd.labshare.cn/cb-dock2/php/blinddock.php/ accessed on 14 May 2026) and GEO (https://www.ncbi.nlm.nih.gov/geo/ accessed on 15 May 2026). All URLs were accessed between 12 and 15 May 2026.
