# Supplementary material for: Impact of cigarette toxicants on sarcopenic obesity: An in silico network toxicology and molecular dynamics study
Source: Tob Induc Dis. 2026 Sep 5;24:10.18332/tid/226887. doi: 10.18332/tid/226887 (PMC13572993; doi:10.18332/tid/226887)
Supplement: Supplementary file 1 [file TID-24-156-s1.pdf]

TID
Tobacco Induced Diseases

Supplementary file

© 2026 Li Z. et al.

DOI:
10.18332/tid/226887

The content has been provided by the author(s) and has not been reviewed, verified, or endorsed by European Publishing. It may not have undergone peer review. The views, opinions, and recommendations expressed are solely those of the author(s) and do not necessarily reflect the position of European Publishing. European Publishing accepts no responsibility or liability for any consequences arising from the use of, or reliance on, this content.

Supplementary Figure S1.

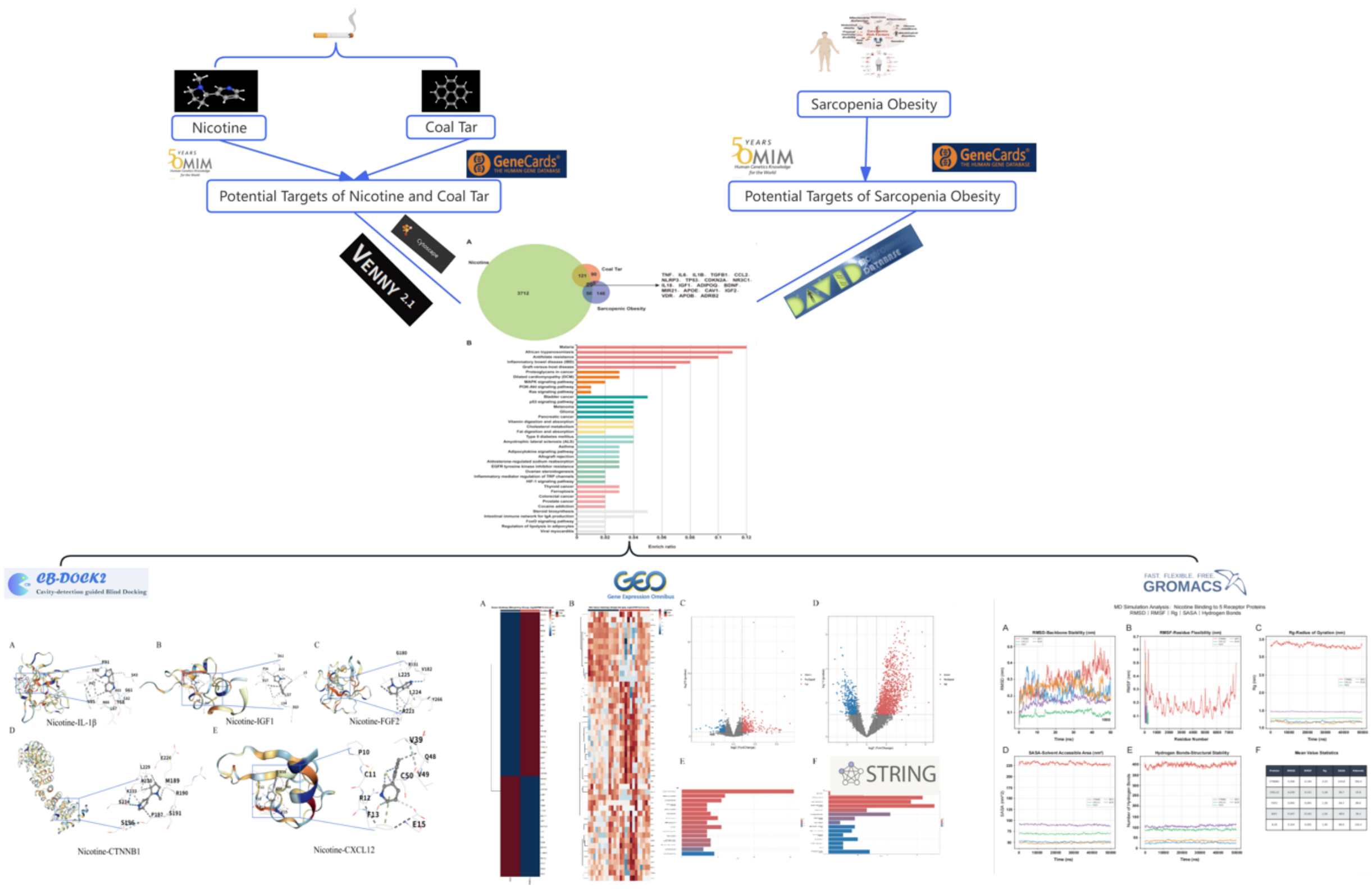

Supplementary Figure S2.

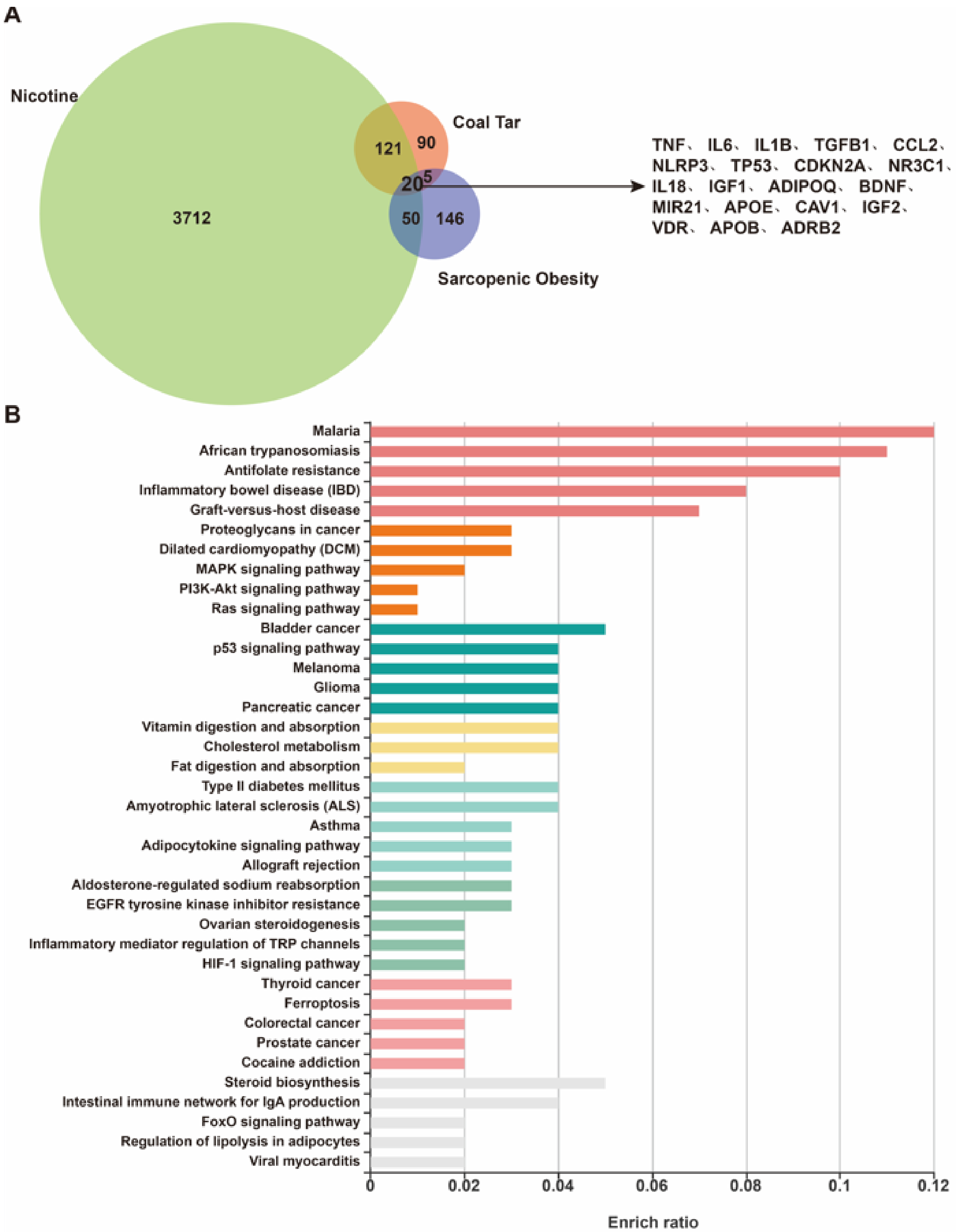

Supplementary Figure S3.

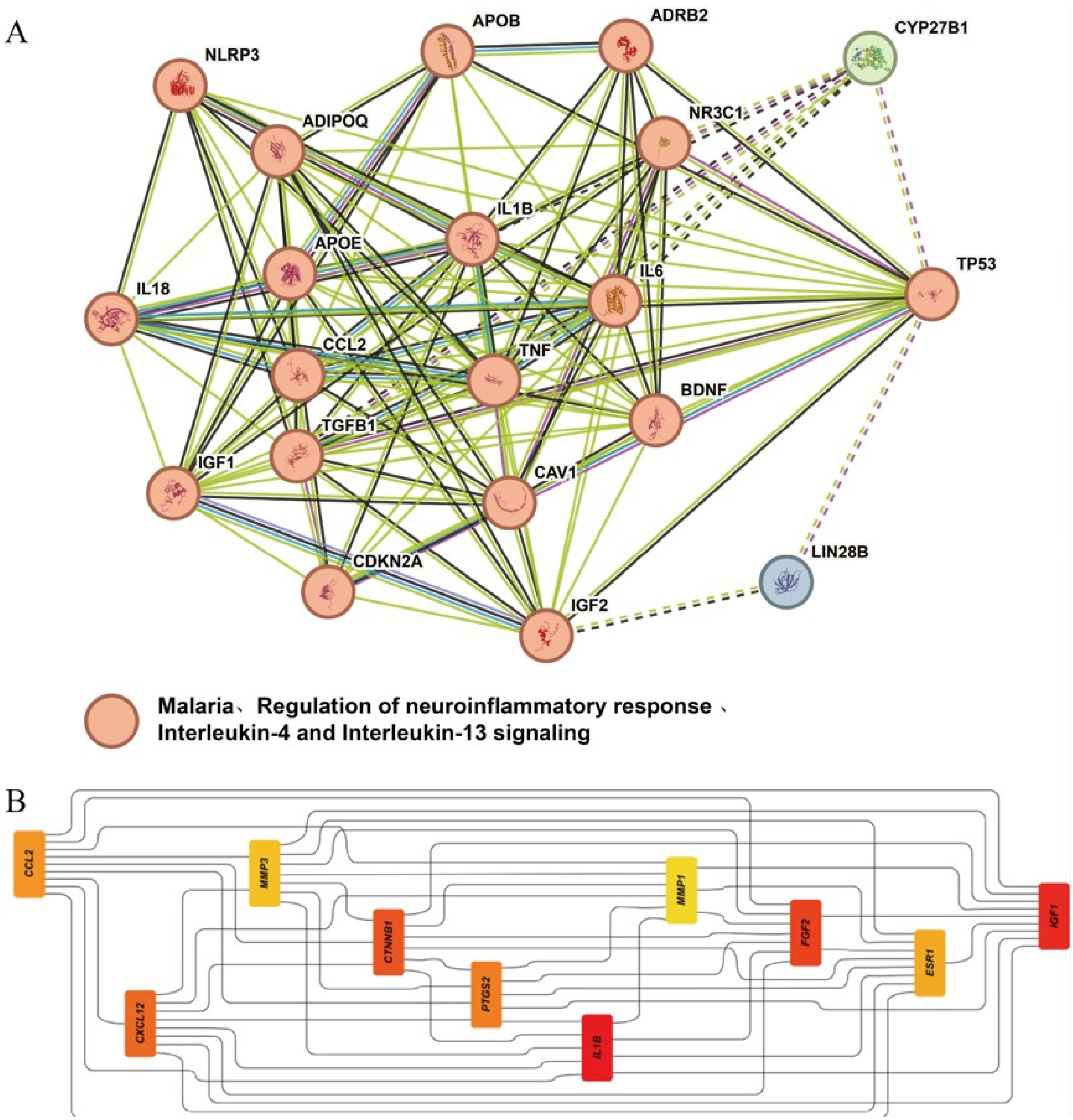

Supplementary Figure S4.

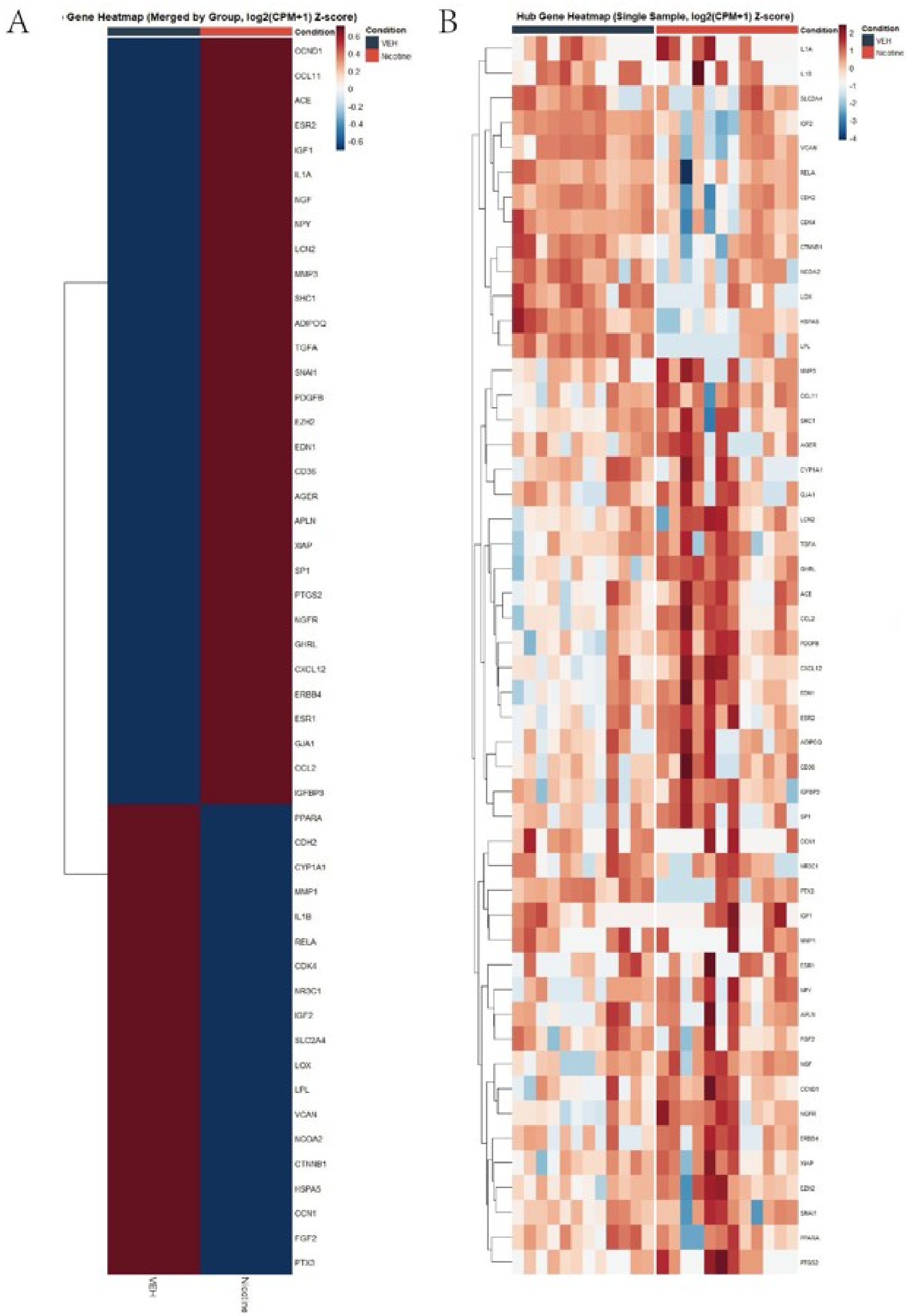

Supplementary Figure S5.

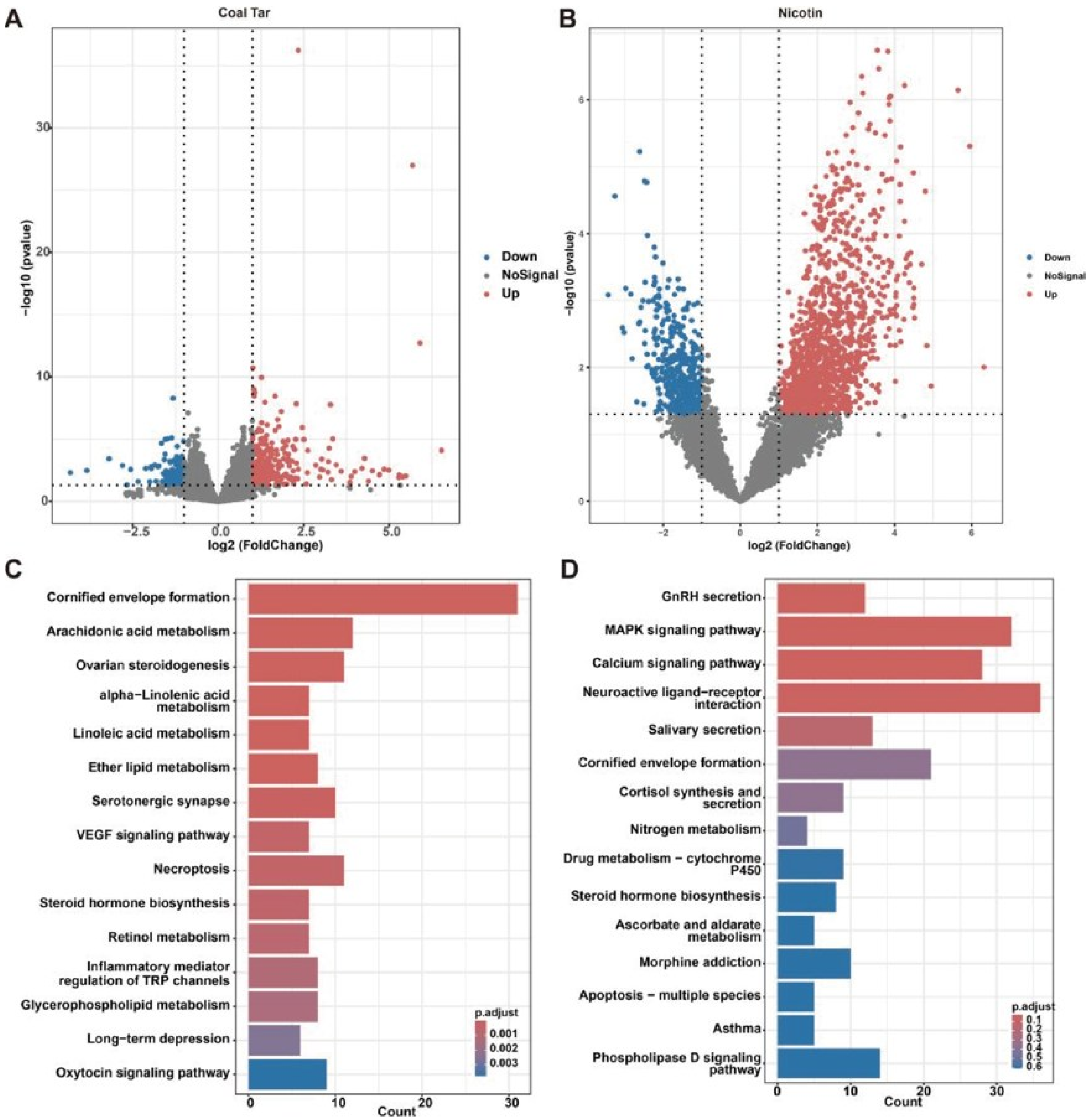

Supplementary Figure S6.

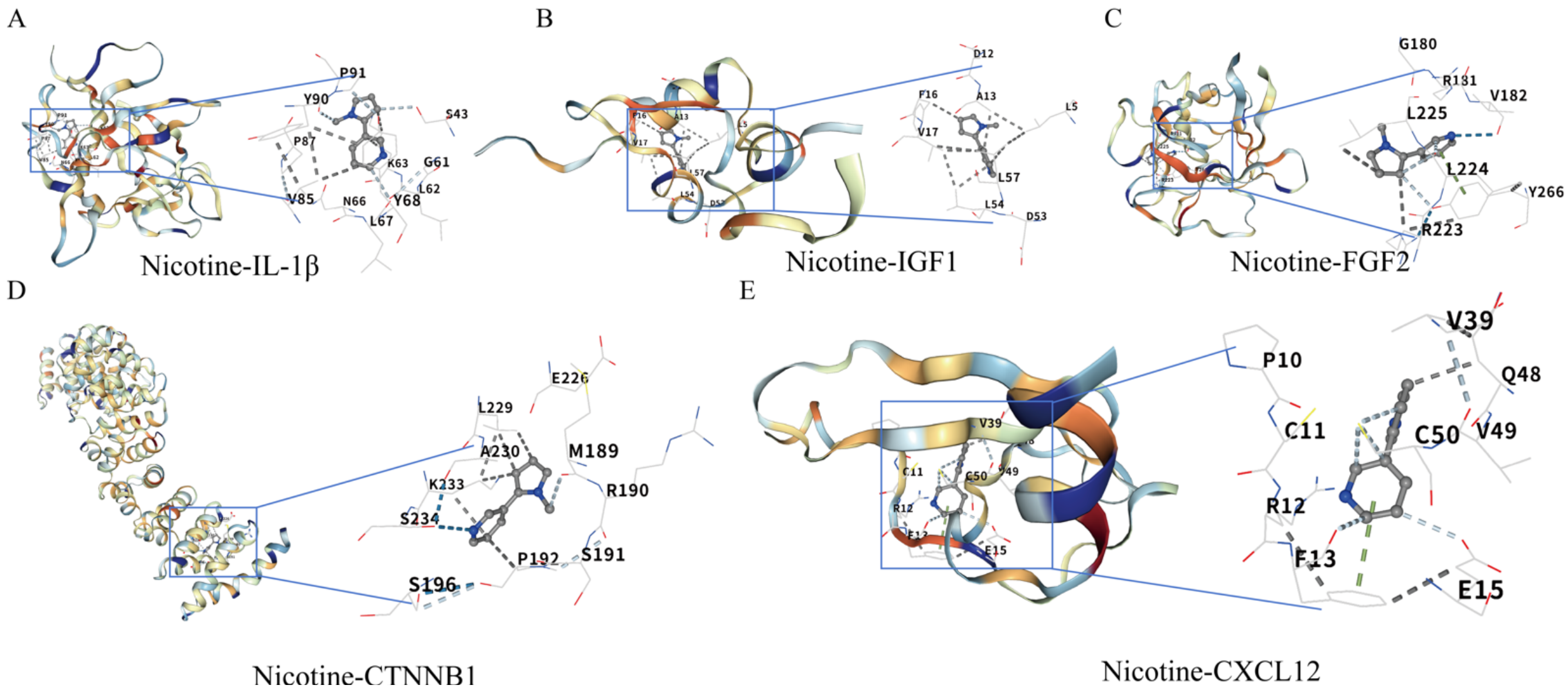

Supplementary Figure S7.

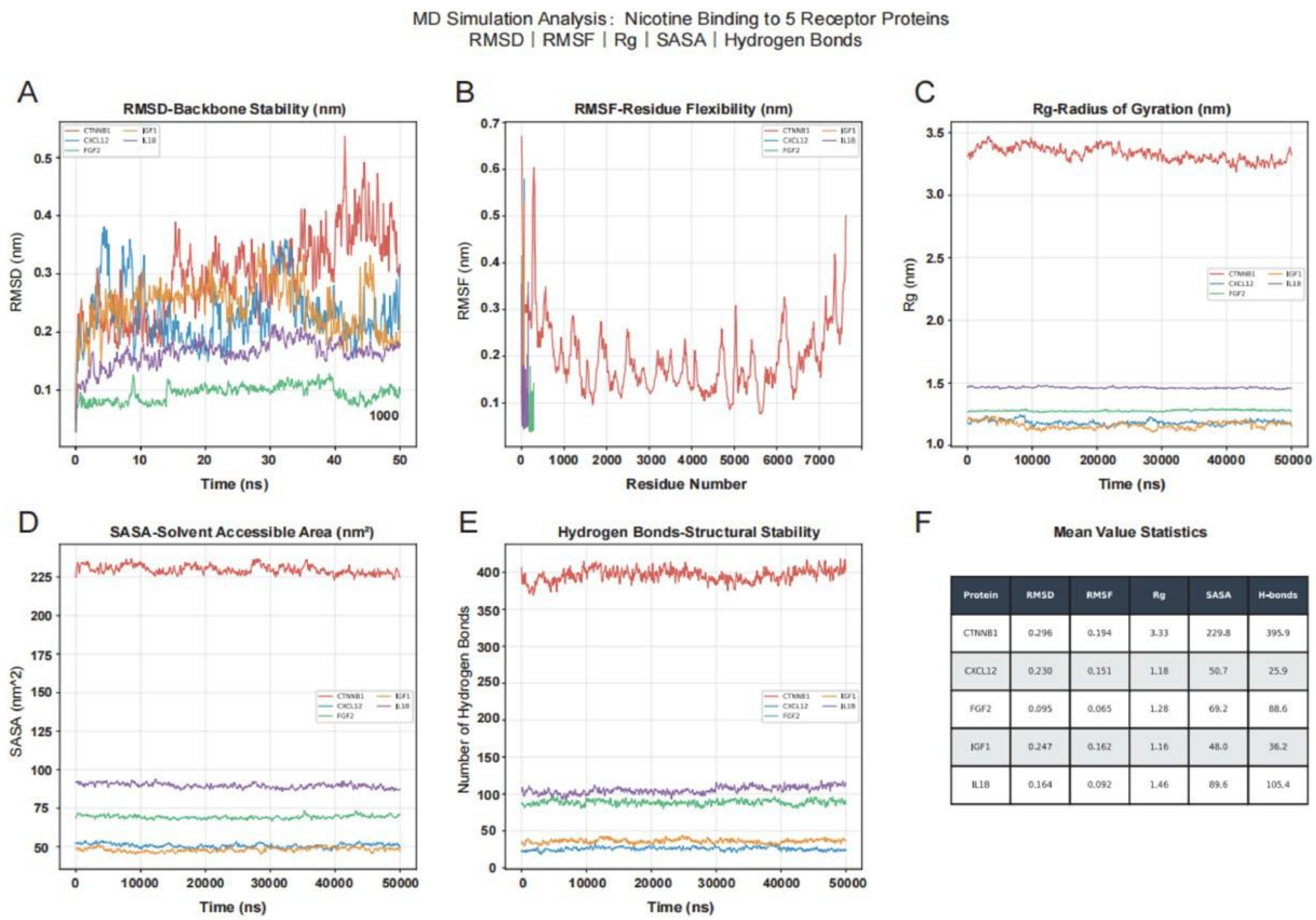

Protein	RMSD	RMSF	Rg	SASA	H-bonds
CTNNB1	0.296	0.194	3.33	229.8	395.9
CXCL12	0.230	0.151	1.18	50.7	25.9
FGF2	0.095	0.065	1.28	69.2	88.6
IGF1	0.247	0.162	1.16	48.0	36.2
IL18	0.164	0.092	1.46	89.6	105.4
